# Pretreatment Embolization of Tumoral Arteriovenous Fistula Associated With Recanalized Umbilical Vein Shunt in a Case of Hepatocellular Carcinoma With Hepatic Vein and Inferior Vena Cava Invasion

**DOI:** 10.7759/cureus.44784

**Published:** 2023-09-06

**Authors:** Joey Almaguer, Ahmed Khan, Arsalan Saleem

**Affiliations:** 1 Radiology, Texas Tech University Health Sciences Center, Amarillo, USA; 2 Vascular and Interventional Radiology, University of Texas Medical Branch, Galveston, USA

**Keywords:** bclc, tare, mapping, y-90, embolization, right hepatic artery, umbilical vein, hepatocellular carcinoma, arteriovenous fistula

## Abstract

Hepatocellular carcinoma (HCC) is the most common liver cancer and has a propensity to develop arteriovenous fistulas with the surrounding vasculature, making targeted intravascular treatment more difficult. HCC can oftentimes be accompanied by portal hypertension and liver cirrhosis, which can, in turn, cause recanalization of the umbilical vein. In rare circumstances, arteriovenous fistula formation and shunting into the recanalized and enlarged umbilical vein can occur. In the following presented case of HCC, an arteriovenous shunt between the anterior division of the right hepatic artery and a recanalized umbilical vein is demonstrated. Subsequent successful endovascular coil embolization of the fistula was performed to avoid shunting and non-target embolization of the radiation particles in the umbilical vein territory. Post-embolization angiogram with DynaCT and lack of Tc-99m macroaggregated albumin deposition in the umbilical vein distribution confirmed the resolution of the shunt. The patient then received targeted Y-90 transarterial radioembolization locoregional therapy in combination with systemic therapy.

## Introduction

Hepatocellular carcinoma (HCC) is the most common primary malignancy of the liver and one of the most common causes of fatal cancers worldwide [1]. There are numerous long-established risk factors that are linked to the development of HCC, such as tobacco abuse, alcohol abuse, and hepatitis B virus (HBV) and hepatitis C virus (HCV) infection. Chronic liver disease and cirrhosis remain the most important predisposing risk factors for the development of HCC [2]. There is a synergistic effect between HCV infection and alcohol abuse, increasing the likelihood of HCC development by 1.7 times [3]. Alpha-fetoprotein (AFP) is the most common tumor marker used for detecting HCC with a specificity close to 100%. However, the sensitivity is less than 45%, with up to 30% of HCC patients having normal AFP levels [4,5].

The Barcelona Clinic Liver Cancer (BCLC) staging system outlines several factors to be considered when determining HCC treatment and prognosis. These factors include AFP levels, albumin-bilirubin (ALBI) score, Model of End-stage Liver Disease (MELD) score, Child-Pugh score, Eastern Cooperative Oncology Group Performance Status (ECOG PS) rating, and individual patient characteristics [6]. Current available therapeutic options include percutaneous local ablation (radiofrequency, microwave, and cryoablation), surgical resection, liver transplantation, stereotactic body radiation therapy, transarterial chemoembolization (TACE) or transarterial radioembolization (TARE), systemic therapies (tyrosine kinase inhibitors, immune checkpoint inhibitors, etc.), and best supportive care [7].

Yttrium-90 (Y-90) radioembolization is a form of TARE that delivers radioactive microbeads laced with Y-90 directly to the HCC tumor through injection from the feeding artery. Y-90 is an unstable radioactive isotope that emits cytotoxic beta particles with a half-life of 64.2 hours and mean tissue penetration of 2.5 mm [8]. The Y-90 resin microspheres become permanently deposited within the arterial supply of the mass, providing both mechanical microembolization as well as radiation damage, ultimately resulting in tumor ischemia and cellular destruction. The purpose of TARE initially began as palliative but has now become a means for downstaging for tumor resection or liver transplantation, or possibly curative treatment, depending on the BCLC stage [9].

Before TARE can be performed, mapping is required for several treatment planning purposes: angiography of the hepatic vasculature, predicting the level of Y-90 pulmonary shunting to avoid ≥30 Gy to the lungs, and determining the appropriate therapeutic Y-90 dosage to avoid ≥40 Gy to the surrounding healthy liver parenchyma [10]. A lung shunt fraction of >20% is considered a contraindication for Y-90 treatment [11]. In select cases where the shunt fraction is limitingly high, the fistulas may be embolized pre-TARE to minimize Y-90 shunting to the lungs.

Although TACE is the more established treatment option, TARE is increasing in popularity for several reasons. In comparison to TACE, TARE appears to be just as efficacious, providing a similar overall survival rate at one-year post-procedure, yet a longer time to progression of HCC [12,13]. TARE can also offer lower rates of post-embolization syndrome and serves as a more suitable therapeutic option for patients with concurrent portal vein thrombosis [14,15]. Lastly, studies have demonstrated that TARE has a better health-related quality-of-life profile in comparison to TACE [16,17]. Some tertiary health care centers have adopted TARE as the primary treatment option for HCC across all BCLC stages [18].

The following presented case describes a rapidly growing BCLC stage B multinodular HCC lesion with an extension of non-occlusive eccentric tumor thrombus into the hepatic veins and hepatic inferior vena cava (IVC) (about 5-7 mm) and with concerns for high lung shunt fraction. In addition and notably, on pre-procedure cross-sectional imaging and mapping angiography, significant shunting into a prominently dilated umbilical vein was noted.

## Case presentation

A 55-year-old male presented with intractable right upper quadrant abdominal pain, abnormal weight loss, decompensated grade IV liver cirrhosis, and intermittent hepatic encephalopathy. Notably, a prior history of two episodes of perihepatic peritoneal tumoral hemorrhage was treated with bland embolization at an outside hospital. He had a past medical history of chronic HCV infection, tobacco abuse (30 pack-years), and alcohol abuse. His AFP marker was elevated at 4,136 ng/mL (normal: <20 ng/mL), with his previous AFP level being 8.4 ng/mL 11 years prior. He was positive for both HCV and HAV antibodies, reflecting his chronic HCV viral infection and either a prior HAV infection or immunization. He was negative for antibody detection against HIV and HBV. Anemia and thrombocytopenia were present and likely related to long-standing liver cirrhosis.

**Figure 1:**
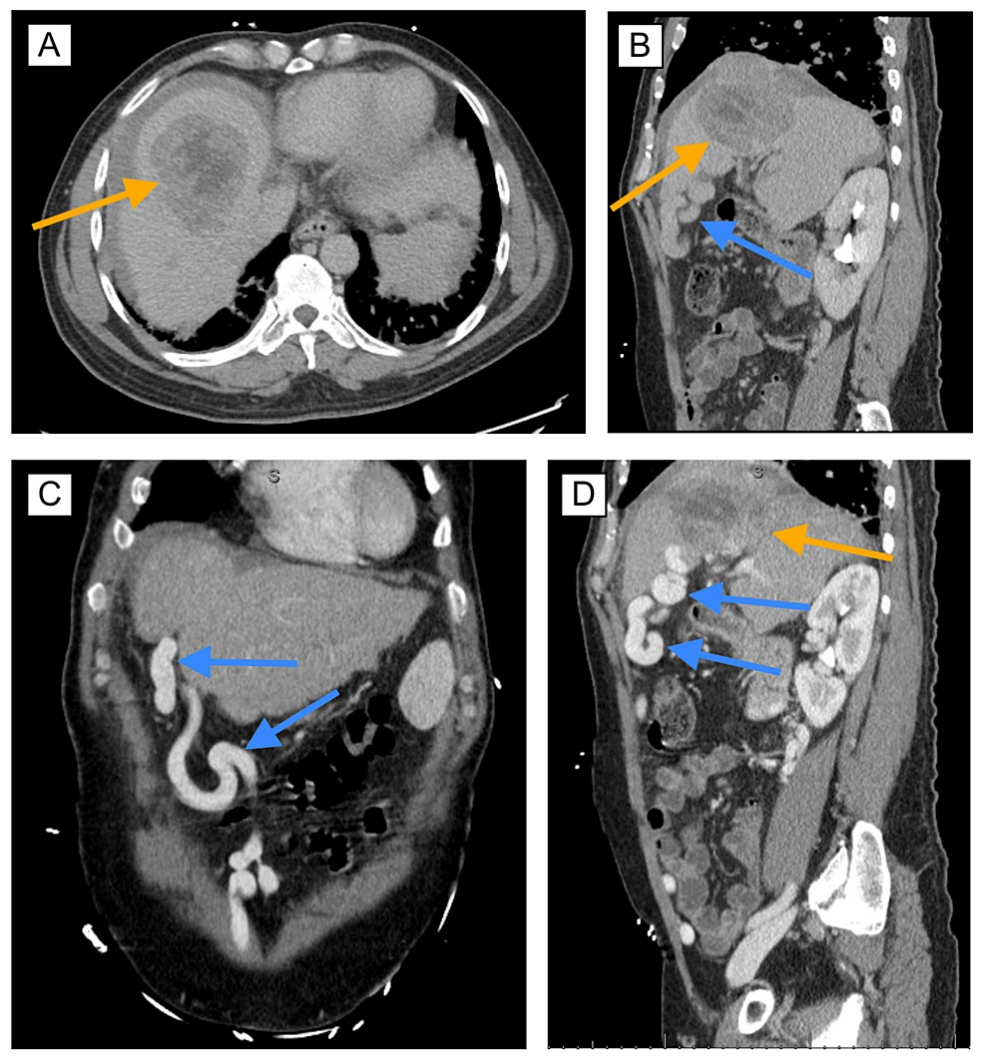
CT imaging of HCC tumor and recanalized umbilical vein. (A/B) CT without contrast showing HCC tumor (orange arrow) and recanalized umbilical vein (blue arrow). (C/D) Venous-phase CT with contrast demonstrating HCC tumor and recanalized umbilical vein. CT: computed tomography; HCC: hepatocellular carcinoma

Subsequent CT imaging revealed two liver masses: a 10 × 7 × 8 cm exophytic hemorrhagic mass in segment 7 and a smaller 2.6 × 1.9 cm mass in segment 8, both of which were highly representative of HCC (LI-RADS category 5) with superior transcapsular subdiaphragmatic extension without peritoneal seeding. There was no active extravasation of contrast into the existing perihepatic hemorrhagic fluid accumulation and no evidence of distant metastasis. The mass was complicated by IVC and right hepatic vein invasion, as well as porta hepatis lymphadenopathy. There was also portal hypertension present, as evidenced by splenomegaly (16 cm), portosystemic collaterals, duodenal varices, a large recanalized umbilical vein, and a small volume ascites (Figure 1). Esophagogastroduodenoscopy revealed banded grade II esophageal varices and portal hypertensive gastropathy. The patient underwent magnetic resonance cholangiopancreatography, which was equivocal.

Pathology revealed that the HCC was stage IIIB (cT4, cN0, cM0). The patient had an ECOG PS score of 0, an ALBI score of -2.5, a MELD-Na score of 15, a MELD score of 13, and a Child-Pugh score of B. These factors determined the HCC in question to be BCLC intermediate stage (B). The recommended treatment options for this BCLC stage included liver transplantation, TACE/TARE, or systemic therapy. The patient was not a candidate for liver transplantation, making TACE/TARE and systemic therapy the therapeutic options available. The case was discussed at the multidisciplinary gastrointestinal tumor board meeting, which determined local Y-90 radioembolization, as well as atezolizumab and bevacizumab combination systemic therapy to be the next course of action. The tumor board recommended the patient start atezolizumab immediately and then begin bevacizumab after the Y-90 treatment procedure had been completed.

**Figure 2:**
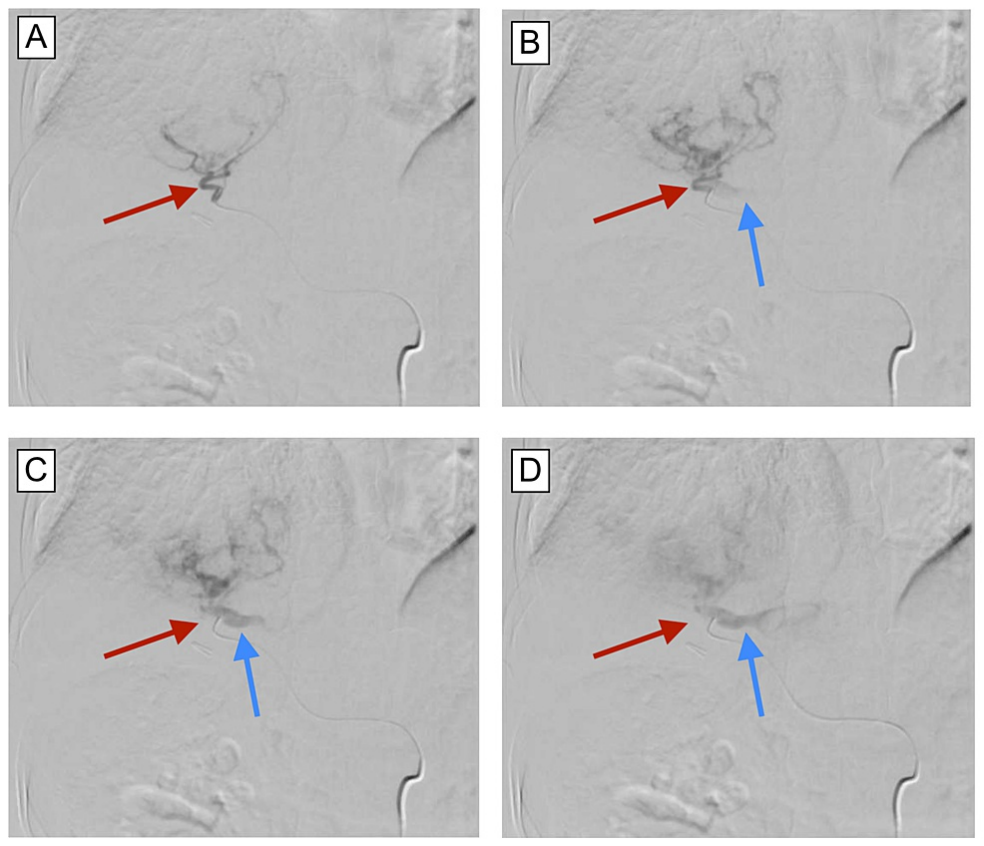
Arterial-to-venous shunting of contrast through umbilical vein fistula. (A) Right hepatic artery (red arrow) opacified with contrast. (B) As contrast distributes, the fistulized umbilical vein (blue arrow) becomes opacified. (C) Increased opacification of recanalized umbilical vein fistula. (D) Maximum opacification of umbilical vein fistula.

With moderate sedation, ultrasound-guided access to the right common femoral artery was obtained using a micropuncture kit. A vascular sheath was inserted into the access site and a Benson wire was then advanced into the abdominal aorta. A Mikaelson catheter was sheathed over the Benson wire and advanced to the level of the celiac artery. Contrast injection confirmed celiac artery catheterization with subsequent angiography. The Benson wire was replaced with a microsystem consisting of a microcatheter and a microwire. Angiography, DynaCT, and three-dimensional (3D) reconstruction performed at the level of the proper hepatic artery revealed a large hypervascular hepatic mass with significant arteriovenous fistulization. The microsystem was advanced to the anterior branch of the right hepatic artery, where angiography revealed an arteriovenous fistula (AVF) formation with shunting to the large recanalized umbilical artery (Figure 2).

**Figure 3:**
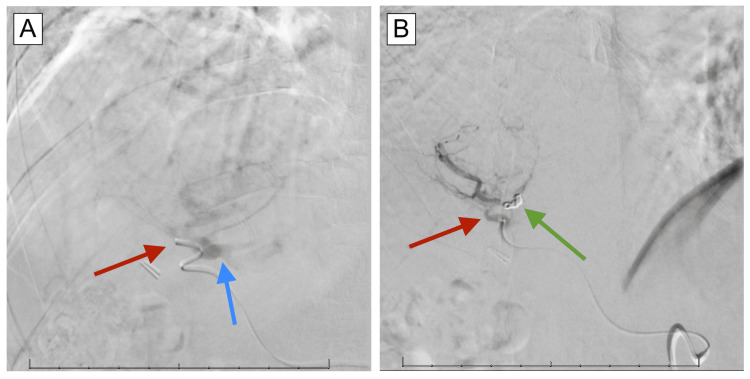
Right hepatic artery angiography before and after coil embolization. (A) Angiography showing arteriovenous shunting of contrast from right hepatic artery (red arrow) to recanalized umbilical vein (blue arrow). (B) Coil embolization (green arrow) of the anterior division of the right hepatic artery prevents shunting of contrast into the umbilical vein fistula.

**Figure 4:**
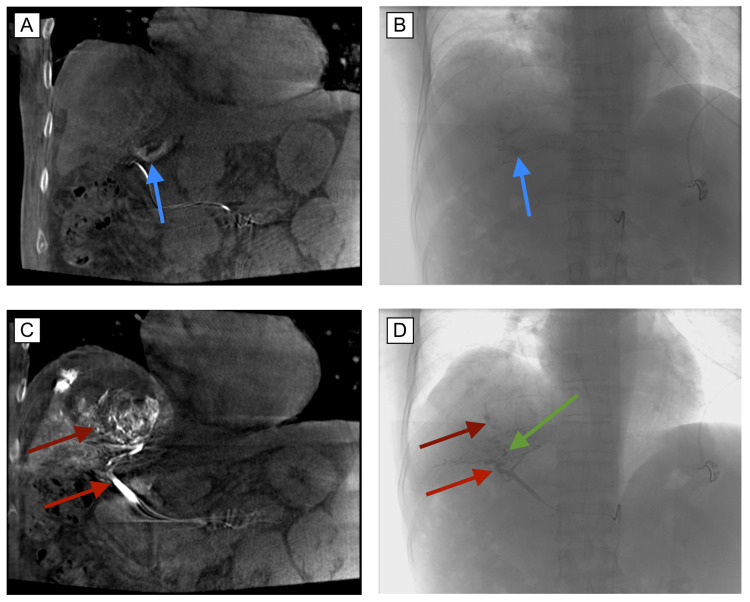
DynaCT before and after coil embolization of umbilical vein fistula. (A/B) DynaCT demonstrating 3D rendering of umbilical vein fistula (blue arrow) before coil embolization. (C/D) DynaCT after coil embolization (green arrow) depicting a lack of blood shunting from right hepatic artery (red arrow) with subsequent increase in perfusion (maroon arrow) to HCC tumor. 3D: three-dimensional; HCC: hepatocellular carcinoma

**Figure 5:**
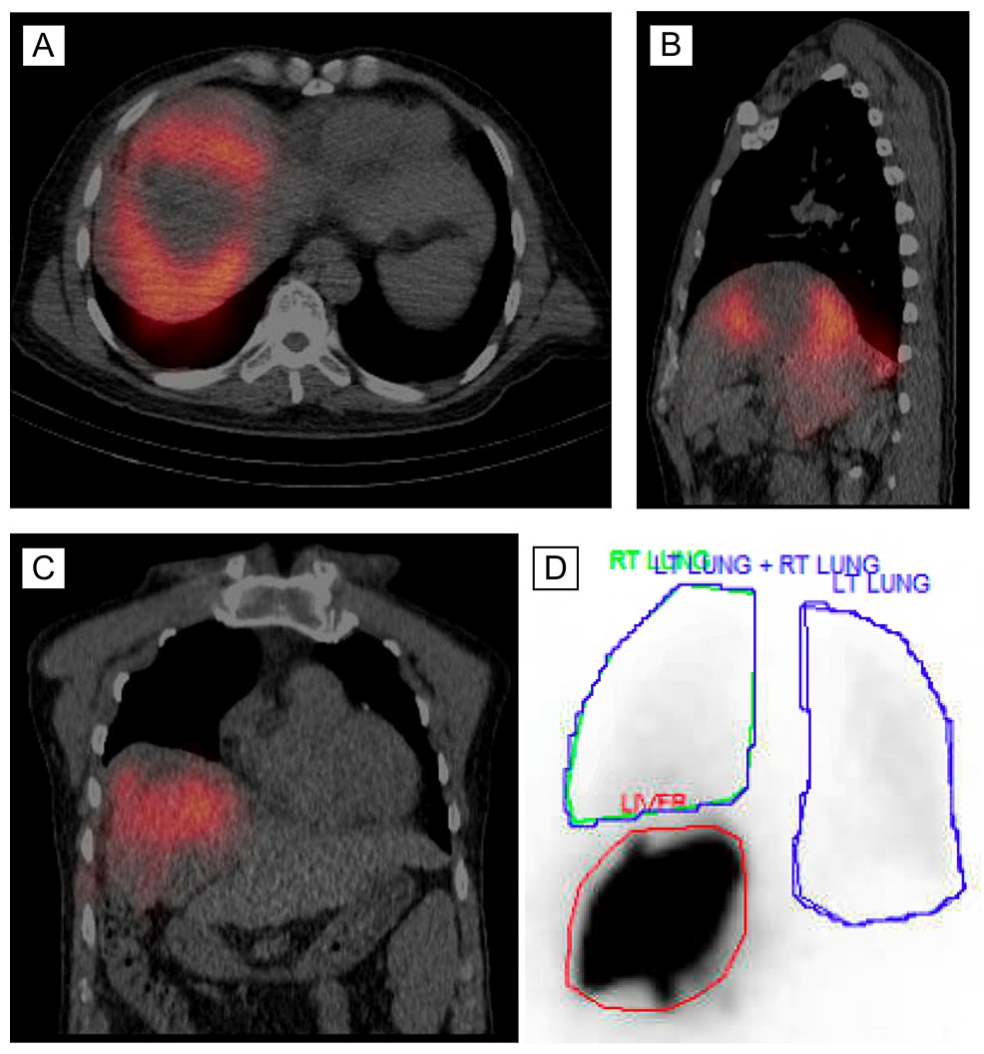
Lung perfusion scan showing distribution of Tc-99m MAA. (A/B/C) Axial, sagittal, and coronal views of Tc-99m MAA distribution throughout the liver. (D) Minimal distribution of Tc-99m MAA into the lungs, with the vast majority remaining in the liver parenchyma. Tc-99m MAA: Tc-99m macroaggregated albumin

Embolization of the unnamed medial-subsegmental branch of the segment 8 artery was performed using metallic coils (Figure 3). Additionally, embolization of the unnamed lateral subsegmental branch of the segment 8 artery was performed using Gelfoam. Post-embolization angiography, DynaCT, and 3D reconstruction at the level of the right hepatic artery demonstrated cessation of anterograde flow in the shunt, as evidenced by a lack of opacification of the recanalized umbilical vein. There was a resultant remodulation of blood flow with increased flow and opacification to the mass (Figure 4). Tc-99m macroaggregated albumin (Tc-99m MAA) was then injected at the site of the right hepatic artery for Y-90 mapping. The common femoral artery arteriotomy site was closed using 6 Fr Angioseal. Subsequent lung perfusion scanning revealed 91.36% of the Tc-99m MAA remaining in the liver and 8.64% traveling to the lungs (Figure 5). There were no complications noted and the patient was discharged the next day with instructions to return the following week for Y-90 treatment.

**Figure 6:**
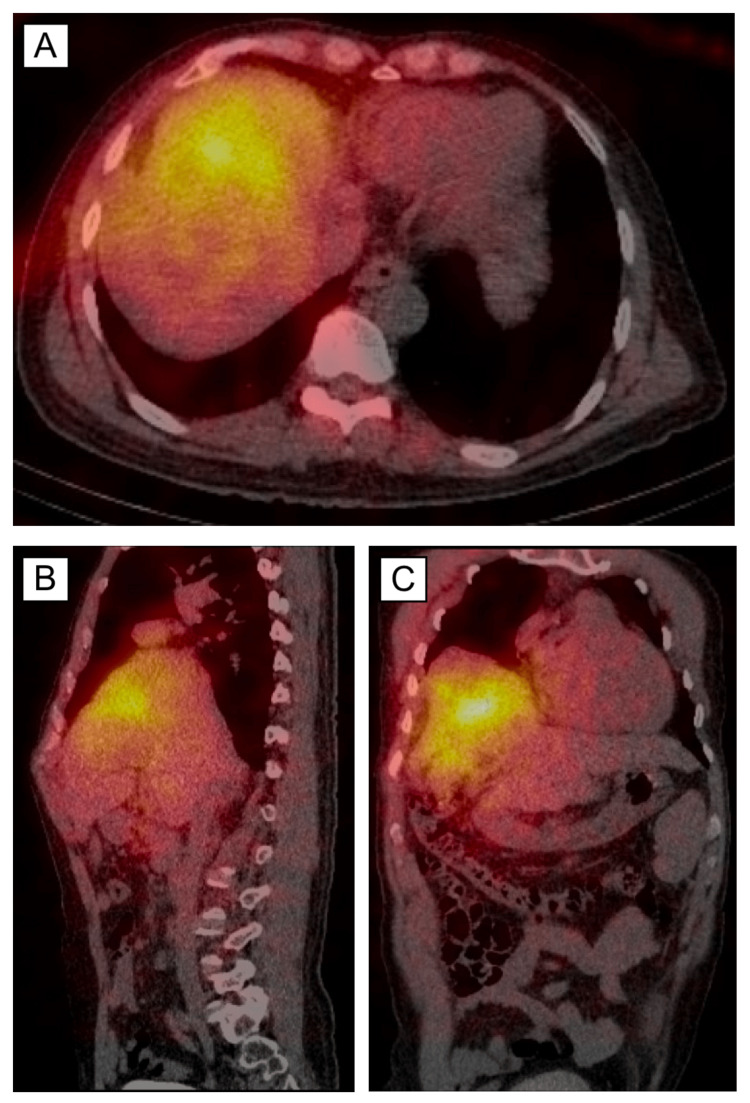
SPECT CT showing Y-90 microembolic microsphere distribution. (A/B/C) Axial, sagittal, and coronal planes showing Y-90 microembolic microspheres that are localized primarily to the liver area of interest where the HCC resides. SPECT: single-photon emission computed tomography; HCC: hepatocellular carcinoma

The mapping Tc-99m MAA single-photon emission computed tomography (SPECT) CT demonstrated significant uptake in the tumor-free hepatic segment 5, relative to the tumoral segment 8, discordant to the intraprocedural DynaCT findings. Hence, on the day of treatment, this segment was embolized with Gelfoam to spare the healthy liver from radiation and increase the blood flow and treatment particle delivery to the tumoral segment 8. Y-90 microembolic microspheres were injected intravascularly at the level of the right hepatic artery with subsequent SPECT CT demonstrating successful uptake of Y-90 microbeads localized to the area of interest (Figure 6). The patient was discharged on the same day and continued to receive his systemic therapy after successful hemostasis of the access site utilizing successful deployment of the arteriotomy closure device.

## Discussion

The umbilical vein is an embryologically significant vascular structure that provides oxygenated blood from the placenta to the fetal circulation in utero. After birth, the umbilical vein begins to involute to form the ligamentum teres hepatis (round ligament of the liver), a fibrous structure connecting the liver to the umbilicus [19]. Under intense portal pressure, this umbilical vein remnant can recanalize and shunt blood into portosystemic collateral vessels around the umbilicus. Umbilical vein recanalization is one of the major signs of portal hypertension, such as esophageal varices [20]. In patients with portal hypertension and liver cirrhosis, 28% were found to have a patent umbilical vein, although this decompressive mechanism does not seem to significantly reduce the severity of portal hypertension [21].

AVF presence is not an uncommon finding in HCC, with one study noting a prevalence of 31.2%: 28.8% involved portal vein shunting, and 2.4% involved hepatic vein shunting, with no report of umbilical vein shunting [22]. Similarly, another study noted no arteriovenous shunting into the umbilical vein among HCC patients undergoing Tc-99m MAA scanning [23]. However, one study of Tc-99m MAA scans saw umbilical vein deposition in five of 159 (3.1%) patients reviewed [24]. This makes the presented case of arteriovenous shunting into a recanalized umbilical vein a rare and unusual finding. To our knowledge, there have only been a handful of papers published in the medical literature outlining AVF formation involving a recanalized umbilical vein [25,26].

In cases where there is HCC extension into the portal vein or hepatic vein, combinatorial treatment involving locoregional therapy and systemic therapy can provide increased response rates [27]. As with most HCC arteriovenous shunts, transarterial embolization remains the preferred intervention when preparing for TARE procedures to prevent non-target Y-90 delivery [28]. It is uncertain whether the systemic distribution of Tc-99m MAA would occur in the setting of a patent umbilical vein AVF or to what degree that distribution would occur. However, to maximize the number of Y-90 micoembolic microspheres that become deposited in the HCC tumor of interest, endovascular coiling of a recanalized umbilical vein AVF can better facilitate optimum treatment results and minimize adverse side effects. The presented case outlines such a scenario where embolization of a recanalized umbilical vein resulted in 91.36% of the Tc-99m MAA remaining in the liver, preventing excessive non-target Y-90 radioembolism.

## Conclusions

Portal hypertension and liver cirrhosis in the presence of HCC resulting in a recanalized umbilical vein is not an entirely uncommon phenomenon. However, HCC AVF formation with a recanalized umbilical vein is a rare occurrence. For patients undergoing targeted liver-directed therapies, including TARE and TACE, an AVF associated with shunting vessels, including portal vein, hepatic vein, and umbilical vein, can have significant side effects if left patent during these locoregional therapies. Hence, endovascular embolization of these AVFs is quintessential for the safe and targeted delivery of Y-90 microembolic microspheres to the HCC with minimal adverse effects. In particular, AVF formation with associated recanalized umbilical vein shunting in the setting of HCC can be recognized as a phenomenon that warrants transarterial embolization to improve patient outcomes.
