# Correction: Pretreatment Embolization of Tumoral Arteriovenous Fistula Associated With Recanalized Umbilical Vein Shunt in a Case of Hepatocellular Carcinoma With Hepatic Vein and Inferior Vena Cava Invasion

**DOI:** 10.7759/cureus.c135

**Published:** 2023-09-14

**Authors:** Joey Almaguer, Ahmed Khan, Arsalan Saleem

**Affiliations:** 1 Radiology, Texas Tech University Health Sciences Center, Amarillo, USA; 2 Vascular and Interventional Radiology, University of Texas Medical Branch, Galveston, USA

This article has been corrected at the request of the authors to change the title from “Pre-transarterial Radioembolization of Tumoral Arteriovenous Fistula Associated With Recanalized Umbilical Vein Shunt in a Case of Hepatocellular Carcinoma With Hepatic Vein and Inferior Vena Cava Invasion” to “Pretreatment Embolization of Tumoral Arteriovenous Fistula Associated With Recanalized Umbilical Vein Shunt in a Case of Hepatocellular Carcinoma With Hepatic Vein and Inferior Vena Cava Invasion”.

The authors and journal deeply regret that this error was not identified and addressed prior to publication.

 

